# Evolution of Cancer Pharmacological Treatments at the Turn of the Third Millennium

**DOI:** 10.3389/fphar.2018.01300

**Published:** 2018-11-13

**Authors:** Luca Falzone, Salvatore Salomone, Massimo Libra

**Affiliations:** ^1^Department of Biomedical and Biotechnological Sciences, University of Catania, Catania, Italy; ^2^Research Center for Prevention, Diagnosis and Treatment of Cancer (PreDiCT), University of Catania, Catania, Italy

**Keywords:** cancer, antineoplastic drugs, chemotherapy, targeted therapy, cell therapy

## Abstract

The medical history of cancer began millennia ago. Historical findings of patients with cancer date back to ancient Egyptian and Greek civilizations, where this disease was predominantly treated with radical surgery and cautery that were often ineffective, leading to the death of patients. Over the centuries, important discoveries allowed to identify the biological and pathological features of tumors, without however contributing to the development of effective therapeutic approaches until the end of the 1800s, when the discovery of X-rays and their use for the treatment of tumors provided the first modern therapeutic approach in medical oncology. However, a real breakthrough took place after the Second World War, with the discovery of cytotoxic antitumor drugs and the birth of chemotherapy for the treatment of various hematological and solid tumors. Starting from this epochal turning point, there has been an exponential growth of studies concerning the use of new drugs for cancer treatment. The second fundamental breakthrough in the field of oncology and pharmacology took place at the beginning of the ‘80s, thanks to molecular and cellular biology studies that allowed the development of specific drugs for some molecular targets involved in neoplastic processes, giving rise to targeted therapy. Both chemotherapy and target therapy have significantly improved the survival and quality of life of cancer patients inducing sometimes complete tumor remission. Subsequently, at the turn of the third millennium, thanks to genetic engineering studies, there was a further advancement of clinical oncology and pharmacology with the introduction of monoclonal antibodies and immune checkpoint inhibitors for the treatment of advanced or metastatic tumors, for which no effective treatment was available before. Today, cancer research is always aimed at the study and development of new therapeutic approaches for cancer treatment. Currently, several researchers are focused on the development of cell therapies, anti-tumor vaccines, and new biotechnological drugs that have already shown promising results in preclinical studies, therefore, in the near future, we will certainly assist to a new revolution in the field of medical oncology.

## Introduction

### Epidemiology of cancer

Cancer is often referred to as the “Pathology of the Century” assuming the connotations of an endemic disease spread throughout the world. It has also been defined as the “the modern disease par excellence” (Roy Porter) or even the “the quintessential product of modernity” (Siddhartha Mukherjee) (Bynum and Porter, 2005; Mukherjee, 2011; Arnold-Forster, 2016). These two definitions are universally recognized and are justified by the drastic increase in incidence and mortality, witnessed since the end of the eighteenth century until today, where cancer represents the second leading cause of death worldwide (Ferlay et al., 2015). In particular, in 2015, over 8.7 million cancer deaths were recorded worldwide and about 17.5 million new cases of neoplasia were diagnosed (GBD Mortality Causes of Death Collaborators, 2016). Moreover, despite advances in the diagnostic, medical and interventional fields, the number of new cases of cancer has increased by about 33% in the decade 2005–2015, mainly due to the increase in population and the increase in the average age of life. Conversely, mortality rates are almost unchanged, although many Countries experienced a decrease in cancer mortality notwithstanding increasing incidence rates (Global Burden of Disease Cancer Collaboration et al., 2018).

**Figure 1:**
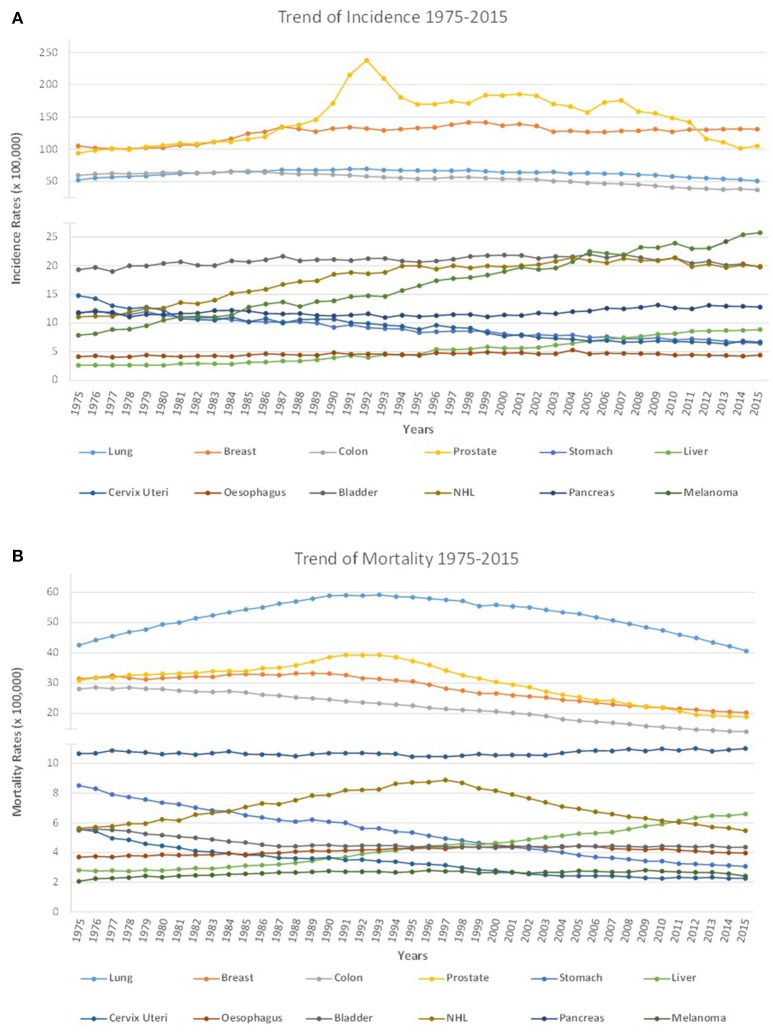
Cancer incidence and mortality from 1975 to 2015. **(A)** In the last 40 years, from 1975 to 2015, there has been a general increase in incidence rates for several cancers as a consequence of the demographic increase. Thanks to cancer prevention and screening strategies the number of many types of cancer has decreased. In particular, for cervix uteri and stomach cancers, a decrease in incidence rates was observed, while the incidence rates for esophagus, bladder, lung and colon remained unchanged. Finally, an increase in incidence rates was recorded for hepatic carcinoma, for breast cancer, NHL and above all for melanoma, whose incidence has increased radically in recent years; **(B)** Cancer mortality rates have globally decreased thanks to the development of targeted therapy and immunotherapy. In particular, in the last 40 years there has been a decrease in mortality rates for prostate, breast, colon, bladder, and especially for cervix uteri and stomach cancers. Almost unaltered mortality rates are observed for melanoma, pancreatic and esophageal cancers for which really effective pharmacological treatments are not yet available. An increase in mortality was observed for hepatic carcinoma while lung cancer and NHL showed a variable trend over the years with a slight increase in mortality rates from 1975 to 1995, and a general decrease in mortality rates recorded above all in the last 20 years.

Supporting these recent epidemiological data, the examination of a longer period of time and the analysis of the data collected by the National Cancer Institute (NCI) over the last 40 years showed that there has been a continuous and almost stable increase in incidence rates of all cancers. While a general decrease in mortality rates was recorded above all in the last 20 years, although from the period between 1975 and 1995 there was a slight increase in mortality rates (Figure 1). The reduction in mortality rates can be easily associated with the continuous progress in the medical and pharmacological fields that has allowed to reduce the cancer deaths, thanks to the recent introduction in therapy of more effective drugs and therapeutic approaches (Soneji et al., 2014; Miller et al., 2016).

When epidemiological data concerning the so-called “big killers” (i.e., cancer of lung, breast, colon, prostate, stomach, liver, cervix uteri, esophagus, bladder, non-Hodgkin's lymphoma (NHL), pancreas, melanoma) are examined, it is instead observed how the trend of incidence and mortality rates are very variable according to the type of pathology taken into account. In particular, some tumor types, such as lung cancer and pancreatic cancer, have still maintained mortality rates almost unchanged compared to 40 years ago (Figure 1).

These epidemiological data show that, even nowadays, cancer represents a global health problem and one of the greatest challenges in the medical field, despite the important pharmacological and therapeutic discoveries we have seen since the second post-war period up to the present day (Gittelman, 2016).

### Cancer treatment before the 1900s

As previously mentioned, cancer is considered a modern disease, but the oncology has their roots in much older times, as evidenced by antique documents dating back to ancient Egyptian and Greek civilizations (Sudhakar, 2009).

Several historical and scientific records show that cancer was present even before the appearance of human on earth. In fact, in some fossil remains of dinosaurs or prehistoric animals traces of bone tumors have been found, probably osteosarcomas or bone metastases (Rothschild et al., 2003; Dumbravbrevea et al., 2016). The first historical and scientific records of tumors in humans date back to the Egyptian period, around 3000 B.C., and refer to the writings contained in a papyrus found by Edwin Smith, in which a case of breast cancer and the surgical treatment adopted were described (Breasted, 1930; Sanchez and Meltzer, 2012).

Other writings date back to 1500 B.C.; in particular, the Ebers' papyrus, contains information on different types of cancer (skin cancer, uterine cancer, stomach cancer and rectum), where the neoplastic pathology is recognized as incurable. In this papyrus, cancer is not considered as due to physical or biological causes, but, rather, as the results of esoteric forces, linked to the negative will of the ancient Egyptian Gods (Ebers, 1875; Bryan and Smith, 1930; Kelly and Mahalingam, 2015).

It was only in 400 B.C. that cancer was recognized as a disease with specific biological causes rather than related to supernatural events. In particular, the first “scientist” describing cancer in a scientific way was Hippocrates, who considered the tumor as a disease caused by the imbalance between the 4 main body humors, i.e., blood, phlegm, yellow and black bile. Furthermore, Hippocrates enunciated the first scientific theory on the origins of cancer, hypothesizing that it originated when there was an excess of black bile in the body, and divided the tumors into three different categories: hard cancers, ulcerated cancers, and hidden cancers, defining the latter incurable (Karpozilos and Pavlidis, 2004; Tsoucalas and Sgantzos, 2016).

The theory of body humors remained predominant for a long time and was further refined by Galen, a physician who lived in Rome around the 130–200 A.C. Galen improved the Hippocratic theory by defining tumors as curable pathologies when caused by the alterations of yellow bile, and incurable when caused by the alterations of the black bile, particularly when this last humor infiltrates the tissues. Furthermore, Galen indicated the first surgical strategies for the treatment of tumors (Galen, 1565; Papavramidou et al., 2010; Hajdu, 2016).

From the Ancient Greek and Greco-Roman Age up to the Modern Era the theories of Hippocrates and Galen remained the most credited. Moreover, from the Egyptian period until the end of the nineteenth century the treatment of cancers was mostly based on the adoption of a healthy diet, cautery and radical surgical approaches for superficial tumor forms, while for the incurable deep forms, mostly used palliative pain therapies, based on poppy (*Papaver somniferum*) extract (Faguet, 2015). The use of these antiquated therapeutic practices often hesitated in the death of the patient, due to the progression of the very same tumor and/or to sequelae of infectious nature related to the surgical intervention and the poor hygienic conditions.

We may consider modern oncology as born in the 1700s, when scientists began to study, for the first time, the carcinogenic effects of some substances, such as tobacco or soot. However, a number of previous important discoveries and inventions paved the way for the birth of modern oncology. From the sixteenth century to the late nineteenth century, there has been a revolution in the medical, surgical and interventional field thanks to the important discoveries of many scientists who studied tumors from an anatomical, biological, epidemiological and therapeutic point of view. This revolution began with the discoveries of Paracelsus (1493–1541) to reach the intuitions of Percival Pott (1714–1788), passing from the invention of the microscope and the theories of cancer onset postulated by Rudolf Virchow (1821–1902), to the first approaches of experimental oncology and radiotherapy promoted by the first medical oncologists and by Marie and Pierre Curie, respectively (Faguet, 2015; see Table 1 for the milestones of oncology before 1900).

**Table 1:** Milestones of oncology research before 1900.

	**Historical period**	**Major discoveries in oncology**
Ancient discoveries and theories of cancer	3000 B.C.	In Edwin Smith's papyrus the first case of human cancer is described
	1500 B.C.	Ebers' papyrus describes the tumors of the skin, uterus, stomach and rectum
	400 B.C.	Hippocrates proposes the first theory on the development of tumors
	130–200	Galen deepens the theory of Hippocrates, proposing that the excess of black bile causes incurable tumors while the excess of yellow bile causes treatable tumors
	300–400	Oribasius of Baghdad confirms that the tumors are caused by an excess of black bile
No significant progress in the study of tumors^*^^,^^**^	527–565	Aëtius of Amida introduces the treatment of breast tumors by amputation of the entire organ
	625–690	Paul of Aegina describes the tumors of the uterus and the surgical approach for the treatment of the bladder, the thyroid and the polypectomy of the nasal polyps
	860–932	Rhazes di Baghdad describes new treatments for tumors in the “De Chirurgia” manuscript.
	980–1037	Avicenna introduces the removal of tumors of the rectum
	1070–1162	Averroes of Cordoba describes the tumors of the esophagus and rectum and introduces the hysterectomy for the removal of uterine tumors
	1500	Paracelsus questions Hippocrates and Galen theories and hypothesizes that tumors develop due to an accumulation of “salts” in the blood
	1543	Andreas Vesalius published the manuscript “De Humani corporis fabrica” containing anatomical information resulting from post-mortem examinations
	1600	Doctors and surgeons propose that the coagulation and fermentation of blood and/or lymph are the cause of the development of tumors
	1600–1620	Invention of the microscope
	1700	Boerhaave hypothesizes that cancer is most likely induced by elements, present in water or in the ground, which defines viruses. It is theorized that chronic inflammation, injury, trauma and family predispositions can determine the development of tumors
	1760	Morgagni hypothesizes that cancer is related to pathological lesions of a particular organ
	1775	Perciaval Pott defines the association between scrotal cancer and exposure to soot in chimney sweeps
	1858	Rudolf Virchow identifies the origin of tumors in the altered cells
	1896	Wilhelm Conrad Röntgen discovers X-rays
Birth of radiotherapy	1896	Emil H. Grubbé uses X-rays to treat breast cancer
	1898	Marie and Pierre Curie discover the radiation emitted by the Radium
	1899	Marie and Pierre Curie suggest using X-rays to treat tumors
	1920	Birth of radiotherapy

* *Hajdu, 2016*;

** *Hajdu, 2017*.

In particular, the origin of radiotherapy dates back to the late XIX century, with the discovery of X-rays by Wilhelm Conrad Röntgen (Röntgen, 1896; Busacchi, 2015). In the following years, Marie and Pierre Curie identified a substance with radiations two million times higher than Uranium (studied by Becquerel), that they called Radium (1898) (Kułakowski, 2011). The two scientists initially studied the use of X-rays for diagnostic purposes but eventually realized that they were harmful at the cellular level, thus suggesting their use in the treatment of tumors (Curie and Curie, 1899). X-rays were already used in 1896 by Emil H. Grubbé for the treatment of breast cancer (Grubbé, 1933; Nakayama and Bonasso, 2016), while Anton Ultimus Sjögren applied this treatment to an epithelioma of the mouth in 1899 (Nakayama and Bonasso, 2016). However, modern radiotherapy only began in 1920, when Claudius Regaud demonstrated that radiation fractionation could be used to treat several human cancers, by reducing the side effects of the treatment itself (Deloch et al., 2016; Moulder and Seymour, 2017).

**Figure 2:**
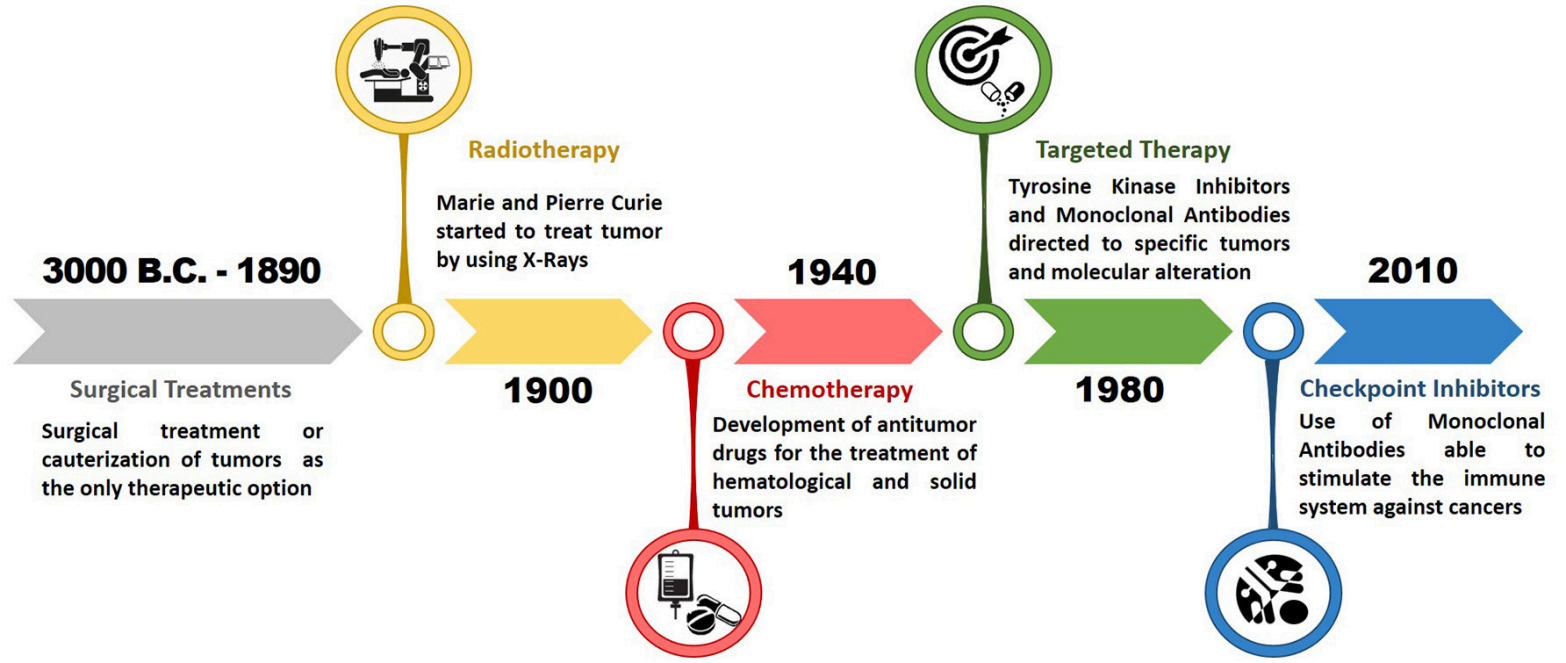
Timeline of epochal turning points in modern oncology. After the development of radiotherapy in the early 1900, the modern oncology began with the discovery of the first chemotherapeutic drugs around 1940. Subsequently a breakthrough in the field of medical oncology occurred with the development of targeted therapy in 1980, which determined an improvement in the effectiveness of cancer treatments. The last epochal turn took place in 2010 with the introduction of immune checkpoints inhibitors for the treatment of advanced and metastatic tumors.

Despite these achievements, medical and interventional approaches to tumors before the Second World War were essentially radical methods, aimed at the complete eradication of the disease before it can spread and metastasize throughout the organism. Therefore, surgical treatment, often representing the only therapeutic option despite its demolition impact, resulted ineffective in patients with advanced tumor pathology or whenever the surgical act failed to remove all the tumor mass (Hajdu, 2011; Hajdu and Vadmal, 2013). An epochal turning point for the treatment of tumors was reached in the mid-1900s, with the birth of chemotherapy and the subsequent evolution of modern medical therapy of tumors. The discovery and synthesis of new compounds represented the basis to develop effective therapeutic interventions in patients with different types of advanced solid tumors or hematological drug treatments, alone or in association with surgical and radio treatments (Arruebo et al., 2011; Figure 2).

The first revolutionary pharmacological approach was represented by the use of chemotherapeutic antitumor drugs, which cytotoxic against various tumors; however, the toxicity to normal tissues and the development of drug resistance mechanisms by tumor cells represented important obstacles to overcome (Chabner and Roberts, 2005).

Subsequently, with the definition of the DNA structure and the development of new molecular techniques for DNA analysis, specific gene alterations responsible for neoplastic transformation were identified. These alterations were studied in order to synthesize drugs specifically targeting them, i.e., the targeted therapies, specific for certain tumors (Krause and Van Etten, 2005).

Furthermore, in the last 20 years new anti-tumor therapeutic strategies, which make use of new biotechnological drugs, have been developed. These strategies have significantly increased the effectiveness of treatments and the survival rates of cancer patients. Among these, monoclonal antibodies and new immunotherapeutic drugs have allowed the development of new personalized therapeutic protocols (personalized medicine) that have shown very high efficacy and low toxicity for patients (Scott et al., 2012; Tsimberidou, 2015). In addition, the research in the field of oncology is constantly aimed at the discovery of new and effective therapeutic strategies, including the promising CAR-T Cell therapy and gene therapy (Yescarta and Kymriah) (Gross et al., 1989; Rosenberg et al., 1990; Vile et al., 2000; Rosenbaum, 2017; Hidai and Kitano, 2018). Moreover, new therapeutic combined protocols, which use different drugs and different types of treatment, are undergoing clinical trials, in order to find therapeutic schemes that can increase the treatment efficacy and reduce the possibility of developing pharmacological resistance (Hu et al., 2010; Vanneman and Dranoff, 2012).

Finally, nowadays, the development of a new drug must necessarily include the integration of multidisciplinary skills to obtain new pharmaceutical molecules available for the market, with good safety and tolerability. In particular, the development of a new drug can no longer be exempt from an initial bioinformatics *in silico* to simulate the level of interaction of hundreds of new molecules with a specific receptor target of the new drug to be implemented. Following the bioinformatics study, it is essential to use several *in vitro* and preclinical animal models to establish the toxicity of the new drug and its therapeutic potential. Therefore, today, bioinformatics and preclinical studies are the fundamental steps to develop a new effective drug endowed with the highest potential efficacy. The *in silico* and preclinical screening of thousands of different pharmacological molecules has in fact allowed the researchers to obtain new oncological drugs which are currently used in clinical practice while significantly reducing mortality from oncological diseases.

## The birth and evolution of chemotherapy for the treatment of tumors

After the discovery and application of X-rays for the diagnosis and treatment of some tumors, there has been a period of standoff for the research of new treatments to be used in cancer care. A new and significant turn to the treatment of tumors took place around the ‘40s of the twentieth century, during the Second World War, with the accidental discovery of the first DNA alkylating agent, a nitrogen mustard derived from iprite, used for war purposes, whose toxic effects determined bone marrow toxicity and killing of white blood cells. In particular, in December 1943, the John Harvey ship carrying nitrogen mustard bombs was bombed and the toxic gas released into the atmosphere; in the following months, almost a thousand men and women previously exposed to the gas died due to complications characterized by bone marrow aplasia (Brookes, 1990).

### Alkylating agents

The bone marrow toxicity of the nitrogen mustard is due to its alkylating activity toward DNA, occurring through two molecular steps; first the aziridinium group of the nitrogen mustard binds the guanine bases, then interstrand cross-links (ICLs) are formed after the displacement of a chlorine (Brookes and Lawley, 1960, 1961). The formation of ICLs is at the basis of the cytotoxic activity of nitrogen mustards, preventing DNA duplication and leading to cell death, particularly in the presence of high cell turnover. Later on, in 1946, Alfred Gilman and Louis Goodman at Yale University discovered the pharmacological effect of nitrogen mustards on organisms affected by certain tumors, such as Hodgkin's lymphoma and other lymphomas and leukemia (Gilman, 1946, 1963). Between 1946 and 1948, the first results of the clinical studies on the therapeutic efficacy of nitrogen mustards were published, formally defining the first chemotherapeutic drugs used in modern oncology (Goodman and Wintrobe, 1946; Rhoads, 1946; Faloon and Gorham, 1948).

The first nitrogen mustard to be used as an alkylating agent in clinical practice was Mechlorethamine, able to bind nitrogen N7 of guanine and to inhibit DNA replication by the above-described mechanisms. In particular, the first uses of Mechlorethamine were intended for patients with prostate cancer and in patients with lymphoid malignancies, such as Hodgkin's disease, lympho-reticulosarcomatosis and lymphatic leukemia (Kieler, 1951; Goodwin et al., 1967). First generation nitrogen mustards are no longer used, due to the high toxicity and pharmacological resistance mechanisms developed by tumor cells. Presently, the nitrogen mustard mainly used in oncological treatments is cyclophosphamide, a bischloroethylenic compound, able to interfere with the cell cycle of both active and quiescent cells (Friedman and Seligman, 1954; Lane, 1959). Although cyclophosphamide can be used in the treatment of various forms of cancer, it is mostly used for the treatment of neoplastic diseases involving the immune system. It is used in the treatment of lymphoma, multiple myeloma, leukemia, ovarian cancer, breast cancer, non-small cell lung carcinoma (NSCLC), neuroblastoma, sarcoma, as well as in the treatment of several autoimmune diseases (Emadi et al., 2009; Brummaier et al., 2013; Kim and Chan, 2017).

Other alkylating agents are represented by nitrosourea compounds (carmustine, lomustine, semustin, streptozocin, nimustine, tallimustine, photemustine), alkyl sulfates (busulfan, treosulfan, mannosulfan), ethyleneimine derivatives (thiotepa, triazichinone), epoxides (etoglucide), triazene compounds (dacarbazine, temozolomide) and metal salts (cisplatin, carboplatin, oxaliplatin, satraplatin) (Puyo et al., 2014). Among these, dacarbazine and platinum compounds are alkylating agents still widely used in the first and second line treatments of various tumors. These agents are used for melanomas, Hodgkin's lymphomas, soft tissue sarcoma, NSCLC, carcinoma of the esophagus, carcinoma of the stomach, bladder cancer, genitourinary tumors, head and neck cancer, ovarian cancer, and carcinoma of the testis (Lokich, 2001; Al-Badr and Alodhaib, 2016).

In particular, dacarbazine was first synthesized by Shealy in 1962 and was approved by the Food and Drug Administration (FDA) in 1975 for the treatment of melanoma and lymphomas (Shealy et al., 1962). The discovery of platinum compounds by Rosenberg and colleagues at the Michigan State University took place in 1965, with cisplatin (first generation), and was further implemented with the synthesis of carboplatin (second generation) and oxaliplatin (third generation), which have revolutionized the treatment of several solid tumors, thanks to their broader antitumor activity and comparatively less nephrotoxicity (Rosenberg et al., 1965; Evans et al., 1983; Rossi et al., 2005).

### Antimetabolites

Soon after the Second World War, new therapeutic approaches for the treatment of tumors have been developed, based on the use of molecules mimicking the structure of physiological metabolites, thereby blocking enzymatic chains essential for the synthesis of purines, which results in inhibition of cell proliferation. The main antimetabolites include folate analogs (aminopterin and methotrexate), purine analogs (mercaptopurine) and pyrimidine analogs (fluorouracil, gemcitabine, capecitabine; Kaye, 1998; Tiwari, 2012).

Antifolates were the first class of antimetabolites studied. In 1947, Sidney Farber, a pathologist at Harvard Medical School in Boston, obtained with aminopterin the first complete pharmacological remission in a child affected by acute lymphoblastic leukemia. Following this observation, aminopterin was the first folic acid analog used to reduce tumor cells proliferation and restore the bone marrow homeostasis (Farber et al., 1948; Thiersch, 1949). The remission of acute pediatric leukemia stimulated the research of other antifolate derivatives, which conserved therapeutic efficacy but exerted less toxic effects. Among the various synthesized compounds, methotrexate (amethopterin), a methylated derivative of endopterin, is still one of the most important currently available antineoplastic drugs (Meyer et al., 1950).

The mechanism of action of both aminopterin and methotrexate was not initially clear. Ten years after Faber's findings, antifolates were shown to specifically inhibit the enzyme dihydrofolate reductase (DHFR). In particular, methotrexate permanently bound DHFR, leading to inhibition of thymidylate and purine synthesis and, subsequently, to the induction of apoptosis (Jolivet et al., 1983). This mechanism of action has proven to be very effective in limiting the tumor growth of numerous solid tumors, including breast, ovarian, head and neck, and bladder cancer (Jolivet et al., 1983). Furthermore, methotrexate has been shown to lead to complete remission of patients with choriocarcinoma and as adjuvant therapy to prevent the onset of osteosarcoma relapse after surgery (Li et al., 1958; Jaffe et al., 1974; Chabner and Roberts, 2005).

In the early '50s, other antimetabolites were synthesized and many of them are still used nowadays. Among these, 6-mercaptopurine and 5-fluorouracil, analogs of purines and pyrimidines, respectively, are widely used in clinical practices for the treatment of both hematological malignances and solid tumors (De Abreu et al., 2000; Wei et al., 2018). In 1954, Skipper and Hitchings studied the purine analogs by developing a drug, 6-mercaptopurine, able to compete with hypoxanthine and guanine for the synthesis of their nucleotide derivatives (Hitchings and Elion, 1954; Skipper et al., 1954). Furthermore, following its conversion into thioinosinic acid (TIMP), 6-mercaptopurine in turn inhibits several enzymatic reactions, including the formation of 5′-adenylic acid (AMP) fundamental for DNA and RNA synthesis. 6-mercaptopurine treatment has been shown to be particularly effective in patients with acute lymphocytic leukemia (ALL) and acute myeloid leukemia (AML).

Finally, the pyrimidine analogs made their entry in cancer clinical practice with the introduction of 5-fluorouracil (5-FU). In 1957, Charles Heidelberger synthesized 5-FU, which has revolutionized the treatment of gastrointestinal tumors with particular reference to colorectal cancer, where 5-FU is still actively used in association with others anticancer drugs in several protocols, such as the FOLFOX and FOLFIRI regimens (Heidelberger et al., 1957; Carrillo et al., 2015).

### Antimitotics of natural origin

The important medical and pharmacological findings obtained in the early '40s and '50s have revolutionized the therapeutic approaches for cancer, leading to significant improvements in survival rate, especially for patients with onco-hematological diseases (Frei, 1985). A new boost to the chemotherapeutic treatment of both hematological and solid tumors has occurred with the introduction in the therapeutic practice of natural extracts with cytotoxic activity, able to interfere with the formation of microtubules and to block the mitotic processes and cell proliferation (van Vuuren et al., 2015). These compounds were commonly classified as microtubule-destabilizing agents or microtubule-stabilizing agents (Chen and Horwitz, 2002) because they act either by inhibiting the polymerization of microtubules via the interaction with the spindle assembly checkpoint (SAC) or by stabilizing microtubules and prevent Ca^2+^- or cold-induced depolymerization, with subsequent blockage of mitotic fuse disassembly (Chen and Horwitz, 2002).

The first antimitotic agents discovered in the late '50s were extracted from the plant *Catharanthus roseus* (rosy periwinkle), and took the name of Vinca alkaloids. Vinca alkaloids were firstly used for the treatment of diabetes, but further studies by Noble and colleagues showed the carcinostatic activity against transplantable mammary adenocarcinoma and sarcoma in mouse models (Noble et al., 1958). Subsequently, in 1963, Johnson and colleagues elucidated the molecular mechanisms underlying the effect of Vinca on tumor cell proliferation (Johnson et al., 1963). In the following years, numerous Vinca derivatives were synthesized, all with depolymerizing action against microtubules. Among these molecules, those with greater therapeutic efficacy were vinblastine, vincristine, vinorelbine, vindesine, etc. (Jordan and Wilson, 2004). All these drugs are still widely used in first and second line therapy for the treatment of various forms of cancer (acute lymphocytic leukemia, malignant lymphomas, multiple myeloma, metastatic breast cancer, small cell lung carcinoma (SCLC), Ewing's sarcoma, embryonal rhabdomyosarcoma, etc.) representing a relevant pharmacological option for patients developing drug resistance to other chemotherapeutic agents. Finally, as described at the end of this chapter, Vinca alkaloids are used in several combined therapeutic regimens.

Other natural antimitotic agents are derived from *Podophyllum peltatum* (mayapple, wild mandrake) from which podophyllin is obtained, initially used for the treatment of epitheliomas and sarcomas with a high toxicity. All *Podophyllum* derivatives, called epipodophyllotoxins, determine the arrest of cell proliferation by blocking the topoisomerase II, fundamental for DNA unwinding during the duplication phase (Imbert, 1998). Given its generalized toxicity, derivatives with higher selectivity and fewer side effects were obtained, including the recently revoked teniposide (VM-26) and etoposide (VP-16) still used in polychemotherapy schedules for the treatment of SCLC, acute monoblastic leukemia and non-seminomatous testis carcinomas (Minocha and Long, 1984).

Finally, the class of antimitotic agents includes topoisomerase I inhibitors (topotecan, irinotecan) and microtubule stabilizing molecules, of which taxanes (paclitaxel, docetaxel, cabazitaxel) represent the most important compounds (Oberlies and Kroll, 2004). In particular, irinotecan is the latest derivative of camptothecin, extracted from an ornamental Chinese tree, *Camptotheca acuminate* (happy tree, cancer tree, or tree of life), in 1966 by Wall and Wani at the Research Triangle Institute (Wall et al., 1966). Irinotecan has been shown to have much more effective antitumor activity than first-generation camptothecins and less renal toxicity. Since 1996, irinotecan has been approved for the treatment of colorectal carcinoma, alone or in combination with 5-fluorouracil or platinum compounds, and subsequently also used for the treatment of NSCLC and ovarian cancer (Rothenberg, 1996; Rosen, 1998).

Five years later, in 1971, the same research group of Wall and Wani described the molecular structure of taxol, a natural compound with antimitotic properties extracted from the tree *Taxus brevifolia* (Pacific yew or western yew, Wani et al., 1971). However, only in 1979, Susan B. Horwitz and her research group described the mechanism of action of Taxol, highlighting its activity as microtubule stabilizer (Schiff et al., 1979). Several drugs have been derived from the Taxol. The progenitor is represented by paclitaxel, still used in clinical practice. Later on, second- (docetaxel) and third-generation (cabazitaxel) derivatives were developed (Bissery et al., 1991; Mita et al., 2009). All these compounds have revolutionized the treatment of several solid tumors including metastatic breast cancer, metastatic pancreatic adenocarcinoma (in association with gemcitabine), NSCLC (in association with carboplatin), head and neck cancer, gastric and prostatic cancer. In particular, these drugs are used when the first line treatment failed in metastatic patients and therefore represent the only therapeutic option for patients who show drug resistance mechanisms or are not candidates for curative surgical interventions (Ojima et al., 2016).

### Cytotoxic antibiotics and related substances

Among the standard chemotherapeutic drugs, there are also some antibiotics and/or their derivatives with marked cytotoxic activity, which are among the most effective anticancer drugs currently used in different therapeutic regimens (Weiss, 1992). A wide range of natural antibiotics displays cytotoxic effects; their main mechanism of action is to form covalent bonds with nucleic acids, interfering with DNA synthesis. The first anti-tumor antibiotic used was puromycin. This is an analog of the adenine capable of integrating within the tRNA molecules on ribosomes and blocking protein synthesis by premature termination of the amino acid chain. However, puromycin was not widely used due to its non-selectivity and high systemic toxicity (Wright et al., 1955).

The discovery of the antitumor properties of the currently used antibiotics in cancer is the result of the active collaboration between European pharmaceutical companies and renowned International cancer research centers (Cassinelli, 2016).

The discovery of anthracyclines is the result of the scientific agreement between the Farmitalia and the Istituto Nazionale dei Tumori in Milan directed by Bucalossi. For the first time, a research center and a company worked together for the discovery and development of a new drug with anticancer properties. For this purpose, in 1960 the constituted workgroup started to study a Streptomyces strain, *Streptomyces peucetius*, found near Castel del Monte (Apulia). A new natural antitumor drug, called daunomycin (in a second moment called daunorubicin), was obtained from this Streptomyces strain and showed higher efficacy compared to others antitumor drugs in patients with chronic lymphoproliferative diseases (Di Marco et al., 1963; Bonadonna et al., 1968). Subsequently, in 1968, a new molecule was extracted from a mutated strain of *Streptomyces peucetius*, obtained by treating the microorganism with N-Nitroso-N-methylurea. This new antitumor drug, named adriamycin, was eventually renamed doxorubicin. The discovery of doxorubicin is the result of the collaborative effort of Farmitalia, the researchers of the Istituto Nazionale Tumori and of researchers at the Memorial Sloan-Kettering Cancer Center in New York. Doxorubicin showed better activity against tumors in mouse and a greater therapeutic index, however, the cardiotoxicity typical of anthracycline was not eliminated (Arcamone et al., 1969; Di Marco et al., 1969). The mechanism of action of anthracyclines consists in the inhibition of DNA and RNA synthesis by interfering with the topoisomerase II enzyme, preventing the relaxing of supercoiled DNA and thus blocking DNA transcription and replication (Hortobágyi, 1997).

Around the mid-‘50s, the anti-tumor antibiotic actinomycin D was obtained from another strain of Streptomyces, *Streptomyces griseus*. This drug was used for the treatment of some sarcomas, choriocarcinoma, and lymphomas. Another anti-tumor drug is the mithramycin belonging to the group of DNA intercalates with high specificity for bone tumors and bone metastases (Kersten et al., 1966).

Finally, another antitumor antibiotic, still widely used in clinical practice today, is bleomycin, discovered in Japan in 1966 and immediately approved for the treatment of various tumors, such as Hodgkin's and NHL, testicular cancer, and cervical cancer, among others (Umezawa et al., 1966; Chen and Stubbe, 2005; Bolzán and Bianchi, 2018).

### Other anti-cancer drugs: polyamine inhibitors and iron-modulating drugs

Cell proliferation and tumor growth are promoted by several stimulating factors including polyamines, organic compounds bearing two or more amino groups, responsible for cell growth, gene transcription, translation, and chromatin remodeling (Miller-Fleming et al., 2015). On these bases, new synthetic drugs were developed in order to prevent the formation of polyamines by inhibiting their synthesis or prevent their transport across the cell membrane. The first polyamine inhibitor, an ornithine decarboxylase inhibitor, was synthesized in the 1970s and was used in clinical practices in 1980 for the treatment of trypanosomiasis and other parasitic infections (Abdel-Monem et al., 1974; Bacchi et al., 1980). Among the ornithine decarboxylase inhibitors, α-difluoromethylornithine (DFMO) is the most widely used both for parasitic infections, excess of facial hair in women, and chemotherapy. DFMO was firstly used in cancer therapy in 1981 for the treatment of kidney and bladder cancer in order to induce the reduction of tumor growth (Dunzendorfer, 1981). Subsequently, numerous studies showed the clinical efficacy of the treatment with DFMO in several cancer types (Gerner and Meyskens, 2004; Damiani and Wallace, 2018). Despite the success of DFMO, DFMO treated cells often up-regulate polyamine transport activity making the treatment ineffective. For this purpose, other molecules, called polyamine transport inhibitors (PTIs), have been produced against polyamine transporters at the cell membrane level. Today these inhibitors are generally used in combination with the DFMO showing that combination therapy is more effective in reducing intracellular polyamine levels, thereby limiting tumor growth (Muth et al., 2014).

Other anti-cancer drugs were developed to regulate the intracellular levels of iron, whose alteration may lead to cancer development. Notably, iron is an essential micronutrient for cellular homeostasis. Iron deficiency is often associated with anemia, while iron increased levels induce oxidative stress to tissues, leading to inflammation, system dysfunction that may lead to genetic alteration and consequently neoplastic transformation. Accordingly, numerous studies tried to develop new iron-modulating agents for the treatment of cancer patients. One of the used iron-modulating anti-cancer drugs is desferrioxamine (DFX), an iron chelator that reduces iron levels and consequently its metabolism, which affects the methylation levels in CRC model (Cao et al., 2018). Among iron chelators, there are also di-2-pyridylketone-4,4,-dimethyl-3-thiosemicarbazone (Dp44mT), ciclopirox, and triapine, for which several clinical trials are undergoing (Fischer-Fodor et al., 2015). In particular, triapine is one of the most used drugs in clinical oncology for the treatment of several solid tumors, including uterine cervix and vaginal cancers, prostate cancer, pancreatic cancer, and advanced/metastatic solid tumors (Fischer-Fodor et al., 2015).

### Combination chemotherapy regimens

During the ‘60s and the ‘70s, new combined therapeutic protocols using several chemotherapeutic drugs with different mechanisms of action began to be proposed in clinical practice.

The use of combined chemotherapy or anticancer polychemotherapy has represented an epochal turning point for the treatment of tumors, because achieves greater therapeutic efficacy than the use of single chemotherapeutic agents. In particular, combination therapy kills a larger number of tumor cells with a higher dose of each single drug, therefore not exceeding the maximum tolerated doses of each single drug. Furthermore, it guarantees a wider range of interaction between drugs and cancer cells with different genetic abnormalities. Finally, it is able to prevent or slow down the subsequent development of drug resistance (Lilenbaum et al., 2005).

In 1964, for the first time, Vincent De Vita and his collaborators, at the National Cancer Institute in Bethesda, proposed a combined approach for the treatment of Hodgkin's lymphoma. This first combination was named MOPP, from the initials of the four antitumor agents used: Mustargen (mechlorethamine), Oncovin (also known as Vincristine); Procarbazine and Prednisone (Moxley et al., 1967). The use of MOPP regimen achieved important therapeutic results, with complete remission in 80% of patients and no signs and symptoms of disease in the following 5 years for 60% of patients (De Vita et al., 1970). This study represented a milestone in the treatment not only for malignant lymphomas, but also for other cancers.

In the following years, thanks to studies carried out in animal models, it was shown that chemotherapeutic drugs were more effective against small tumors and when used in combination, thus establishing the importance of early diagnosis and early treatment of tumors in both adjuvant and neoadjuvant regimens. Based on these studies, new combined treatments were proposed. In June 1972, Gianni Bonadonna and Umberto Veronesi proposed a study to evaluate the efficacy of an adjuvant chemotherapy after surgery, based on the combination of three drugs Cyclophosphamide, Methotrexate, and Fluorouracil, named CMF, which improved the probability of survival of cancer patients (De Lena et al., 1973). In 1973, Bonadonna and some of his collaborators proposed a new combination of four drugs, Adriamycin, Bleomycin, Vinblastine, and Dacarbazine, named ABVD after their initials, for the treatment of Hodgkin's lymphoma. The results demonstrated that, even years later, the ABVD combination healed more patients than MOPP alone and was better tolerated, with minor side effects (Bonadonna et al., 1975).

These first studies on the efficacy of combination therapy have paved the way for the development of numerous therapeutic regimens, still used today, that have proven to be more effective than treatment with individual antitumor drugs. In the following years, the clinical importance of the relationship between the dose intensity and therapeutic efficacy of the administered drugs was confirmed and the first therapeutic protocols based on bone marrow transplantation in leukemic patients were developed.

The discoveries in the field of molecular biology gave a major impulse to develop new targeted therapies and new selective biological drugs, specific for certain tumors. These discoveries prompted the second pharmacological revolution in cancer therapy, that began in ‘80s, with the development of selective kinase inhibitors and monoclonal antibodies.

## The revolution of targeted therapy: selective kinase inhibitors and monoclonal antibodies

At the beginning of the ‘80s, the new discoveries in the field of immunology, cell biology, and molecular biology allowed the researchers to investigate the molecular mechanisms responsible for the neoplastic transformation of cells and thus identify new molecular targets to be blocked by small selective inhibitory molecules or monoclonal antibodies. In particular, unlike the classic chemotherapy approach, which acts on both normal cells and cancer cells, the targeted therapy intervenes on altered key oncogenes or tumor suppressor genes involved in tumor promotion. By this way, these new selective inhibitors are able to affect only altered cancer cells with minor side effects toward the normal cells (Hartmann et al., 2009).

**Figure 3:**
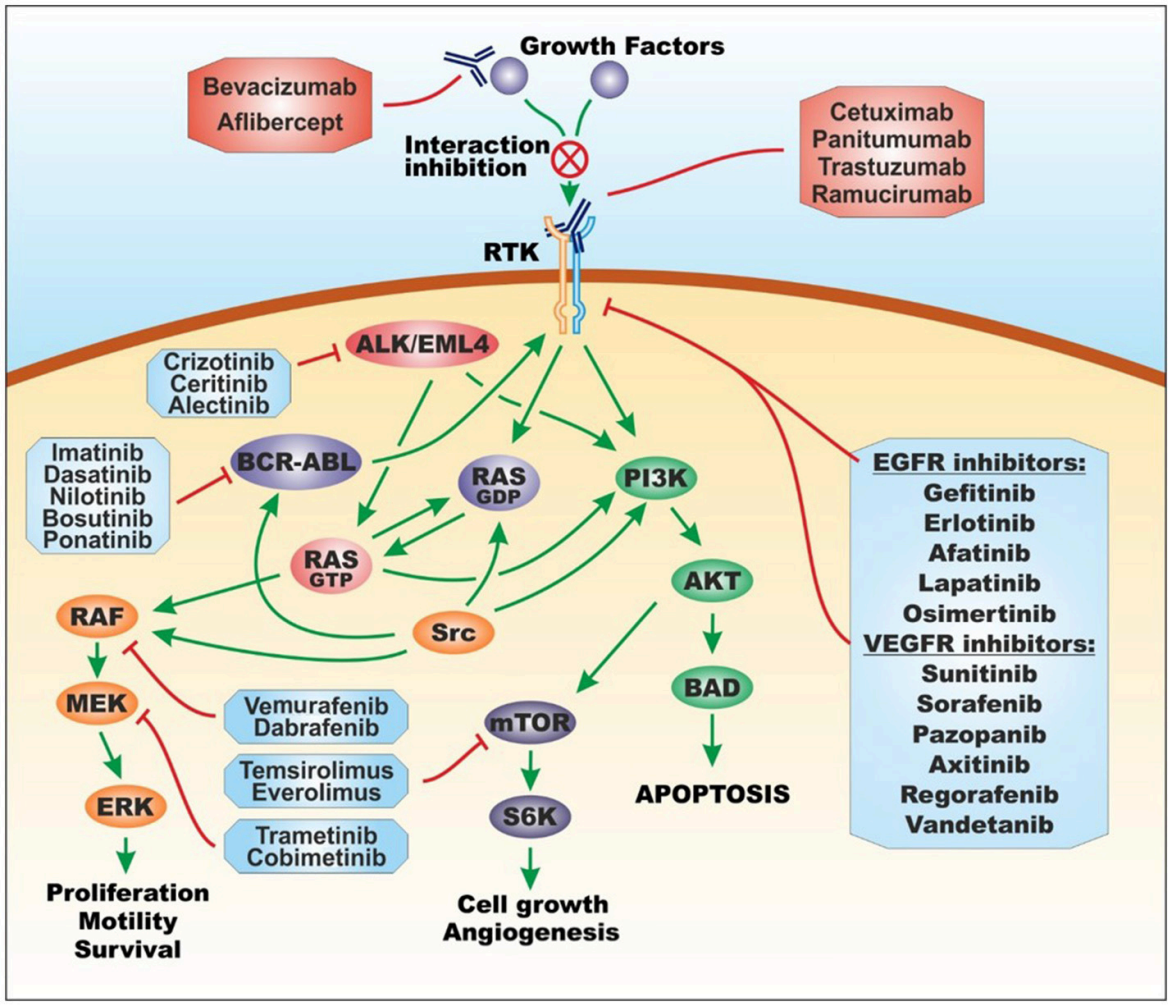
Molecular targets of targeted therapy. Targeted therapy for cancer treatment is based on tyrosine and serine/threonine protein kinase inhibitors and monoclonal antibodies. Protein kinase inhibitors are divided into EGFR inhibitors, VEGFR inhibitors, BCR/ABL inhibitors, ALK/EML4 inhibitors, RAF inhibitors, MEK inhibitors, and mTOR inhibitors. Monoclonal antibodies are directed toward extracellular growth factors or extracellular receptor tyrosine kinase. Figure 3 has been adapted and enriched by taking a cue from two published papers by Massimo Libra, co-author of the present review (Russo et al., 2014; Leonardi et al., 2018). For the general structure of Figure 3 and the name of drugs, the information contained in the book “Farmacologia: Principi di base e applicazioni terapeutiche” was taken into account (Rossi et al., 2016). ABL, Abelson murine leukemia viral oncogene homolog; AKT, protein kinase B; ALK, anaplastic lymphoma kinase; BAD, Bcl-2-associated death promoter; BCR, breakpoint cluster region; EGFR, epidermal growth factor receptor; EML4, echinoderm microtubule-associated protein-like 4; ERK, extracellular signal–regulated kinases; MEK, mitogen-activated protein kinase; mTOR, mammalian target of rapamycin; PI3K, phosphoinositide 3-kinase; RAF, rapidly accelerated fibrosarcoma kinase; RAS, RAS proto-oncogene GTPase; RTK, receptor tyrosine kinase; S6K, S6 kinase; src, proto-oncogene tyrosine-protein kinase Src; VEGFR, vascular endothelial growth factor receptor.

Nowadays, the term “targeted therapy” refers to all those treatments affecting specific molecular targets; this approach takes advantage of either small molecules obtained by chemical synthesis or biological drugs (also called biotechnological drugs), i.e., recombinant proteins, mainly monoclonal antibodies, directed toward specific cellular receptors and proteins involved in neoplastic processes (Tsimberidou, 2015; Figure 3).

The discoveries of 1975 by George Köhler and César Milstein have opened the way for the production of several specific hybrid monoclonal antibodies specific to different antigens or cellular targets, obtained by the realization of hybridomas, resulting from the fusion of murine B lymphocytes and human myeloma cells, capable of producing large quantities of monoclonal antibodies (Köhler and Milstein, 1975).

The first targeted approach to the treatment of cancer dates back to the early ‘80s, with the development of a monoclonal antibody tested on murine models (Bernstein et al., 1980). In 1980, Nadler and colleagues treated a patient affected by NHL with the murine monoclonal antibody AB89, but the treatment did not induce a significant clinical response (Nadler et al., 1980). Nevertheless, this was the first attempt of targeted therapy by using a monoclonal antibody able to selectively target tumor cells and to induce cell death by direct or indirect mechanisms. These two mechanisms of action are respectively defined as the direct inhibition of a molecular pathway involved in tumor progression or the enhancement of host defense mechanisms through activation of the antibody-dependent cytotoxic pathway and complement-mediated cytotoxicity (Oldham, 1983; O'Mahony and Bishop, 2006).

Other attempts to develop effective monoclonal antibodies against myelo- and linfo-proliferative diseases and lymphomas have taken place over the ‘80s, but without results convincing enough to justify their use in clinical practice (Miller et al., 1982). In particular, since most antibodies were murine, they induced host immune reactions against the administered drug, with subsequent side effects (allergic/anaphylactic reactions) and enhanced clearance/reduced half-life (Dillman et al., 1986; Kornbrot et al., 1994).

It was only in the ‘90s that the first really effective targeted therapy drugs became available. The breakthrough was made possible thanks to the studies on the human genome, and the advancement in technologies for DNA sequencing, genomics, transcriptomics and proteomics, invaluable for recognizing new molecular targets (Tsimberidou, 2015). Moreover, in those years, thanks to the new knowledge in molecular and cellular fields and the advancement of the technologies for drug discovery, we have witnessed the birth of modern targeted therapy and personalized medicine. As described below, the possibility of having effective and specific drugs against growth factors and their receptors, cytoplasmic proteins, and signal transducers altered in specific tumor pathologies lead to significant improvement in therapeutic efficacy and survival rates of cancer patients.

### Monoclonal antibodies in cancer therapy

Since the discovery of Trastuzumab (Herceptin®) and the first clinical trials performed in 1992, several monoclonal antibodies were discovered and introduced in cancer clinical practice. As previously mentioned, Köhler and Milstein (1975) revolutionized anti-cancer therapeutics with the development of hybridoma technology, used to produce monoclonal antibodies. Initially, the produced monoclonal antibodies were mouse antibodies. Subsequently, thanks to the new genetic engineering techniques, it was possible to obtain different types of monoclonal antibodies used for the treatment of both hematological and solid tumors. In particular, there are 4 types of monoclonal antibodies available: murine, chimeric, humanized and human monoclonal antibodies, which differ each other by the percentage of murine protein portion present in the immunoglobulin (Pento, 2017).

According to the last report of the “Animal Cell Technology Industrial Platform” (ACTIP), in 2017, 30 different monoclonal antibodies were approved by FDA and/or EMA for the treatment of hematological and solid tumors and others will be approved in the near future; others 6 antibodies were approved for the diagnosis of cancers (Table 2; ACTIP, 2017).

**Table 2:** Monoclonal antibodies approved by the EMA and FDA for cancer treatment and diagnosis −2017 Update.

**Trade name**	**Active principle name**	**Company**	**Target**	**Type**	**Year of EU EMA approval**	**Year of FDA approval**	**Therapeutic indication(s)**
Bavencio®	Avelumab	Merck Sharp & Dohme Limited	PD-L1	Human IgG1/κ	Not approved^*^	2017	Metastatic Merkel cell carcinoma
Imfinzi®	Durvalumab	Astrazeneca UK	PD-L1	Human IgG1/κ	Not approved^*^	2017	Metastatic urothelial carcinoma
Lartruvo	Olaratumab	Eli Lilly	PDGFR-α	Human IgG1	2016	2016	Sarcoma
Darzalex®	Daratumumab	Janssen-Cilag	CD38	Human IgG1/κ	2016	2015	Multiple myeloma
Empliciti	Elotuzumab	Bristol-Myers Squibb	SLAMF7	Human IgG1	2016	2015	Multiple myeloma
Portrazza	Necitumumab	Eli Lilly	EGFR	Human IgG1	2016	2015	Carcinoma, non-small-cell lung
Tecentriq®	Atezolizumab	Genentech (Roche)	PD-L1	Human IgG1	Not approved^*^	2016	Metastatic non-small cell lung cancer
Opdivo	Nivolumab	Bristol-Myers Squibb Pharma	PD-1	Human IgG4	2015	2015	Carcinoma; non-small-cell lung carcinoma; renal cell Hodgkin disease melanoma
Unituxin	Dinutuximab	United Therapeutics Europe	GD2	Human IgG1/κ	2015 (1)	2015	Neuroblastoma
Blincyto®	Bevacizumab	Amgen Europe	CD19	BiTEs	2015	2014	Precursor cell lymphoblastic leukemia-lymphoma
Keytruda®	Pembrolizumab	Merck Sharp & Dohme Limited	PD-1	Human IgG4	2015	2014	Melanoma
Cyramza	Ramucirumab	Eli Lilly	VEGF	Human IgG1	2014	2014	Stomach neoplasms
Perjeta®	Pertuzumab	Roche	HER2	Humanized IgG1	2013	2012	Breast cancer
Gazyvaro®	Obinutuzumab	Roche	CD20	Humanized IgG1	Not approved^*^	2013	CLL
Vervoy®	Ipilimumab	BMS	CTLA-4	Human IgG1	2011	2011	Melanoma
Xgeva®	Denosumab	Amgen	RANKL	Human IgG2	2011	2011	Prevention of SREs in patients with bone metastases from solid tumors
Arzerra®	Ofatumumab	Genmab and GSK	CD20	Human IgG1	2010	2009	Chronic lymphocytic leukemia
Removab®	Catumaxomab	Fresenius	EpCAM and CD3	Trifunctional MAb IgG2a/IgG2b	2009	Not approved^*^	Malignant ascites in patients with EpCAM-positive carcinomas
Vectibix®	Panitumumab	Amgen	EGFR	Human IgG2	2007	2006	Metastatic colorectal carcinoma
Proxinium®	Catumaxomab	Viventia (Eleven Biotherapeutics)	EpCAM	Humanized MAb	2005	2005	Head and neck cancer
Avastin®	Bevacizumab	Genentech (Roche)	VEGF	Humanized IgG1	2005	2004	Metastatic colorectal cancer; non-small cell lung cancer; metastatic breast cancer; glioblastoma multiforme; metastatic renal cell carcinoma
Erbitux®	Cetuximab	ImClone (Eli Lilly), Merck Serono and BMS	EGFR	Chimeric IgG1	2004	2004	Head and neck cancer; colorectal cancer
Campath®	Alemtuzumab	Millennium Pharmaceuticals and Genzyme	CD52	Humanized IgG1	2001	2001	B-cell chronic lymphocytic leukemia
Herceptin®	Trastuzumab	Genentech (Roche)	HER-2	Humanized IgG1	2000	1998	Breast cancer; metastatic gastric or gastroesophageal junction adenocarcinoma
Rituxan® MabThera®	Rituximab	Biogen Idec, Genentech (Roche)	CD20	Chimeric IgG1	1998	1997	Non-Hodgkin's lymphoma; chronic lymphocytic leukemia; rheumatoid arthritis
**CONJUGATED MONOCLONAL ANTIBODIES FOR CANCER TREATMENT**							
Zevalin®	Ibritumomab tiuxetan	Biogen Idec	CD20	Murine IgG1	2004	2002	Non-Hodgkin's lymphoma
Bexxar®	Tositumomab and iodine 131 tositumomab	Corixa and GSK	CD20	Murine IgG2a	Not approved^*^	2003	Non-Hodgkin's lymphoma
Mylotarg®	Gemtuzumab ozogamicin	Wyeth	CD33	Humanized IgG4/toxin conjugate	Not approved^*^	2000 (2)	Acute myeloid leukemia (AML)
Kadcyla®	Trastuzumab emtansine	Roche	HER2	Humanized IgG1 as ADC	2013	2013	Breast cancer
Adcetris®	Brentuximab	Seattle Genetics	CD30 + MMAE	Chimeric IgG1 as ADC (antibody drug conjugate)	2012	2011	Hodgkin lymphoma (HL), systemic anaplastic large cell lymphoma (ALCL)
**DIAGNOSTIC MONOCLONAL ANTIBODIES FOR CANCER**							
Humaspect®	Votumumab	Organon Teknica	Cytokeratin tumor-associated antigen	Human Mab + 99mTc	1998 (3)	Not approved^*^	Detection of carcinoma of the colon or rectum
LeukoScan®	Sulesomab	Immunomedics	NCA90	Murine Fab fragment	1997	Not approved^*^	Diagnostic imaging for osteomyelitis
CEA-scan®	Arcitumomab	Immunomedics	Human CEA	Murine Fab fragment	1996 (4)	1996	Detection of colorectal cancer
ProstaScint®	Capromab	Cytogen	Tumor surface antigen PSMA	Murine MAb	Not approved^*^	1996	Detection of prostate adenocarcinoma
Verluma®	Nofetumomab	Boehringer Ingelheim, NeoRx	Carcinoma-associated antigen	Murine Fab fragment	Not approved^*^	1996	Diagnostic imaging of small-cell lung cancer
OncoScint®	Satumomab	Cytogen	TAG-72	Murine MAb	Not approved^*^	1992	Detection of colorectal and ovarian cancers

* *Not yet approved by EMA or FDA; (1). Withdrawn from use in the European Union; (2). Withdrawn from the market in US in 2010; (3). Withdrawn from the market in EU in 2003; (4). Withdrawn from the market in EU in 2005*.

Both EMA and FDA have released various regulatory guidelines concerning development, clinical experimentation, approval and subsequent commercialization of monoclonal antibodies used in anti-tumor therapies. In particular, in September 2017 EMA released the fifth version of the “Guideline on the evaluation of anticancer medicinal products in man” providing the guidance for the development of anti-cancer drugs, including monoclonal antibodies, in all stages of their clinical development (European Medicine Agency, 2016, 2017). According to EMA, the development of a monoclonal antibody is a multi-step process composed by *in vitro* non-clinical studies performed to elucidate the prime activity of the drug and subsequent pre-clinical studies in animal models and clinical trials in tumor patients to assess the pharmacokinetics, clearance, activity and response to monoclonal antibody treatments (European Medicine Agency, 2016, 2017). Regarding FDA regulations, a document containing all the guidance for the production and regulation of monoclonal antibody drugs was published in 1997, “Points to Consider in the Manufacture and Testing of Monoclonal Antibody Products for Human Use” (Food Drug Administration, 1997). Subsequently, several drafts were approved and published by FDA, but the 1997 document still remains the the main reference text for development and production of new monoclonal antibodies used in oncology and for the treatment of other diseases.

As mentioned above, Trastuzumab was the first monoclonal antibody tested in a clinical trial (1992) directed toward the mutated HER2/neu receptor of breast cancer (Carter et al., 1992). However, the approval of this drug by the regulatory agencies took place only in 1998 (Miller, 1998). Because of this delay, the first approved monoclonal antibody was Rituximab (Rituxan® and MabThera®), approved in 1997, a year earlier than Trastuzumab. The introduction of both Rituximab and Trastuzumab have represented landmark events in the revolution of anti-tumor treatments.

In particular, the first experiments on Rituximab were carried out in 1994 by IDEC Pharmaceuticals Corporation, which, inspired by the studies conducted on murine monoclonal antibodies against the surface antigen CD-20, developed a chimeric monoclonal antibody, named IDEC-C2B8, able to determine the killing of B-cells in both monkeys and B-cell lymphoma patients (Maloney et al., 1994; Reff et al., 1994). Subsequently, in 1997, FDA gave the final approval for the use of this antibody also in patients affected by lymphoproliferative B lymphocyte disorders.

After the approval, all the phase II and III clinical trials showed the promising effects of Rituximab in the treatment of several types of refractory NHLs, including mantle cell and diffuse large B-cell lymphomas (Coiffier et al., 1998). Others studies showed that high doses of Rituximab were well-tolerated by patients affected by indolent NHL and were able to induce the remission of pathology in a high percentage of patients (Ghielmini et al., 2004; Hainsworth et al., 2005). Furthermore, in 2002, a combination therapy including Rituximab and CHOP standard chemotherapy (Cyclophosphamide, Hydroxydaunorubicin (also called doxorubicin), Oncovin (vincristine), and Prednisone or Prednisolone) was proposed (Coiffier et al., 2002; Mounier et al., 2003). The so-called R-CHOP regimen has shown a significant outcome improvement for patients affected by NHLs. Another antibody directed to the CD-20 receptor is Tositumomab, a murine IgG2a lambda monoclonal antibody, produced in mammalian cells. This monoclonal antibody was eventually conjugated with iodine 131 (Iodine I 131 Tositumomab). Both labeled and unlabeled Tositumomab are approved for the treatment of Rituximab-refractory NHLs (Quackenbush et al., 2015).

Regarding Trastuzumab, it is a humanized monoclonal antibody obtained by genetic engineering technologies, able to inhibit the activation of human epidermal growth factor receptor 2 (HER2)/neu, a glycoprotein receptor with tyrosine kinase activity, which, when altered, promotes breast cancer cells growth. Beside receptor blockade, Trastuzumab is able to induce cancer cell death by Antibody-Dependent Cell-Mediated Cytotoxicity (ADCC), a key mechanism also exploited by other monoclonal antibodies (Hudes et al., 2007). Trastuzumab was obtained by Ullrich and Shepard at UCLA's Jonsson Comprehensive Cancer Center (Shepard et al., 1991). Subsequent collaboration between Genentech and UCLA scaled up the development of Trastuzumab that was used in the first clinical trial in 1992 (Carter et al., 1992).

The results obtained by the first phase I and II trials suggest that Trastuzumab, through the repression of the HER2/neu receptor, reduced the aggressiveness of breast cancer cells. Furthermore, these studies showed that Trastuzumab was effective when used in monotherapy as well as in combination with platinum compounds (Baselga et al., 1996; Pegram et al., 1998). The effectiveness and safety of Trastuzumab have made it the gold standard treatment for women with metastatic breast cancer, alone or in combination with paclitaxel or doxorubicin, and for patients affected by HER2-positive metastatic gastric carcinoma (Müller et al., 2018). Finally, in 2013 a conjugated monoclonal antibody was approved, named Trastuzumab-emtansine. This is an antibody-drug conjugate composed by the monoclonal antibody Herceptin chemically linked with the antimitotic agent emtansine (DM1 or mertansine; Niculescu-Duvaz, 2010; LoRusso et al., 2011). This drug showed a higher efficacy compared to Trastuzumab alone because while the monoclonal antibody inhibits the cell growth through its interaction with the HER2/neu receptor and subsequent inhibition of both MAPK and PI3K/AKT cellular signaling pathways, emtansine entries the cell and binds to tubulin, preventing the duplication of DNA (Barok et al., 2014). The first clinical trials showed that Trastuzumab-emtansine significantly improves patient's progression-free survival (PFS) (14.2 months, compared to 9.2 months for patients treated with the standard regimen Trastuzumab plus docetaxel; Hurvitz et al., 2013).

The HER2/neu receptor is also the target of another humanized monoclonal antibody, Pertuzumab (Perjeta®), used in patients affected by HER2-positive metastatic and non-metastatic breast cancer. Pertuzumab is often administered in association with Trastuzumab and docetaxel in adjuvant and neoadjuvant regimens (Schneeweiss et al., 2013). Pertuzumab was discovered and developed by Genentech and then approved in 2012. Similar to Trastuzumab, Pertuzumab is a HER dimerization inhibitor; in particular it prevents the dimerization of HER2 with other HER receptors thus inhibiting intracellular phosphorylation events, which subsequently blocks the abnormal cell growth and proliferation (Harbeck et al., 2013).

The third monoclonal antibody directed to surface receptors discovered in order of time is Cetuximab (Erbitux®) directed against the epidermal growth factor receptor (EGFR), frequently altered in numerous tumors, especially colorectal carcinomas, NSCLC and head and neck cancers (Vokes and Chu, 2006). Cetuximab is a chimeric (mouse/human) monoclonal antibody with a 5- to 10-fold higher affinity for EGFR compared to the endogenous ligands. Its mechanism of action consists in the inhibition of EGFR signaling transduction pathway resulting in the block of cell cycle progression, angiogenesis, cell migration and invasion; furthermore, Cetuximab is able to promote ADCC cytotoxicity and to induce EGFR internalization, resulting in down-regulation of EGFR itself (Vincenzi et al., 2010). Since 1988, Sela and collaborators have studied a monoclonal antibody directed to EGFR and observed a significant therapeutic effect in cellular and pre-clinical models of human carcinomas (Aboud-Pirak et al., 1988). Later on, thanks again to the support of a pharmaceutical company, Cetuximab entered clinical development and showed immediately important results in of phase II and III clinical trials carried out on patients affected by colorectal carcinomas, NSCLC and head and neck cancers (Robert et al., 2001; Kim, 2004; Saltz et al., 2004). This treatment, however, is effective only in the subset of patients without activating mutation of KRAS gene (Lièvre et al., 2006).

In the following years, another human monoclonal antibody, Panitumumab (Vectibix®), directed to EGFR was developed. In 2006, Panitumumab was approved for the treatment of metastatic colorectal cancer patients with wild-type KRAS and refractory to standard chemotherapeutic regimens (Poulin-Costello et al., 2013).

The monoclonal antibodies described above, with the exception of Rituximab directed against the CD-20 differentiation cluster, are all directed to extracellular receptors responsible for the activation of various molecular pathways that have, as their final effect, the increase in cell proliferation and/or inhibition of apoptosis (Fauvel and Yasri, 2014). Another monoclonal antibody widely used for the treatment of different types of solid tumors is Bevacizumab (Avastin®) a recombinant humanized monoclonal antibody directed to a soluble growth factor and not to a receptor. Indeed, Bevacizumab blocks angiogenesis by inhibiting vascular endothelial growth factor A (VEGF-A) representing the first anti-angiogenic factor to be developed (Chen et al., 2001).

The discovery and development of Bevacizumab began with studies by Senger in 1983, who for the first time identified the vascular endothelial growth factor responsible for neovascularization observed in rodents' tumors (Senger et al., 1983). Subsequently, in 1989 the research group directed by Napoleone Ferrara purified and cloned VEGF (Ferrara and Henzel, 1989), starting the development of Bevacizumab (Presta et al., 1997).

The discovery of Bevacizumab and its use in clinical practice represented an epochal turning point for the first and second line treatment of numerous metastatic solid tumors, for which no effective treatments were yet available. The first clinical trials (1997) showed that bevacizumab, used as a single agent, was well-tolerated by patients and when administered in combination with other chemotherapeutic agents did not lead to an increase in systemic toxicity (Gordon et al., 2001). Further phase II and III clinical studies demonstrated the efficacy of Bevacizumab, administered alone, for patients with renal cell carcinoma, or, in combination with standard chemotherapy, for patients with colorectal carcinoma and NSCLC (Ferrara et al., 2004).

Today Bevacizumab is used for the treatment of metastatic colorectal carcinoma, advanced or metastatic breast cancer, advanced metastatic lung cancer, advanced and/or metastatic renal carcinoma, epithelial ovarian carcinoma, fallopian tubes carcinoma, peritoneal carcinoma and recurrent or metastatic cervix carcinoma (Keating, 2014).

Other antibodies used for the treatment of tumors are: Brentuximab (Adcetris®), Ofatumumab (Arzerra®), Alemtuzumab (Campath®), Obinutuzumab (Gazyvaro®), Elotuzumab (Empliciti) and Daratumumab (Darzalex®) for the treatment of hematological malignancies (REF); Avelumab (Bavencio®), Durvalumab (Imfinzi®); Olaratumab (Latruvo), Necitumumab (Potrazza), Atezolizumab (Tecentriq®), Dinutuximab (Unituxin), Ramucirumab (Cyramza), Denosumab (Xgeva®), Catumaxomab (Removab®) for the treatment of several solid tumors (Table 2).

Furthermore, some of these monoclonal antibodies are used for diagnostic purposes (Zhang et al., 2014) and others have been conjugated with cytotoxic molecules or radioactive isotopes, to specifically direct their high toxic activity against tumor cells (Beck et al., 2017; Table 2).

Other monoclonal antibodies developed since 2011 and directed against immune checkpoint inhibitors (Ipilimumab, Nivolumab, Pembrolizumab) will be discussed in more detail in the following chapter on immunotherapy.

### Selective tyrosine kinase and serine/threonine-protein kinase small molecules inhibitors

As stated before, several molecular pathways are altered in cancer because of gene mutations and protein modifications that lead to the abnormal activation of intracellular signal transduction, such as MAPK and PI3K/Akt/mTOR, resulting in the increase of cell proliferation, reduced apoptosis, cell dedifferentiation, and cell migration (McCubrey et al., 2010). The study of these molecular alterations has led to the development of chemical small molecules able to selectively bind to molecular targets present in the tumor cells, causing their inhibition and cancer cell death by apoptotic mechanisms.

The selective inhibitors are generally divided into two main categories: selective tyrosine kinase inhibitors and intracytoplasmic serine/threonine kinase inhibitors (Wu et al., 2015). The new targets include growth factors, signaling molecules, cell-cycle proteins, modulators of apoptosis and molecules that promote angiogenesis. At the beginning of the '90s there was a growing industrial and scientific interest in developing new selective drugs for specific molecular targets known to be involved in cancer development; such growing interest has fostered both the efficacy of new cancer treatments and the economic development of pharmaceutical companies engaged in the development of anticancer drugs (Lange et al., 2014; Diaby et al., 2015).

The landmark event in the revolution of targeted therapy was represented by the development in the early '90s of the first selective tyrosine kinase inhibitor, Imatinib mesylate (Glivec®), a specific competitive inhibitor of ATP, directed to the fusion protein BCR-ABL typical of patients with chronic myelogenous leukemia (CML) and ALL that are Philadelphia chromosome-positive (Ph+). Before the discovery of Imatinib several compounds were obtained for the inhibition of BCR-ABL tyrosine kinase, among these Tyrphostins and similar compounds that displayed limited specificity (Anafi et al., 1993; Waller, 2018). Subsequently, in 1996, Buchdunger and colleagues synthesized a tyrosine kinase inhibitor selective against the ABL tyrosine kinase domain called 2-phenylaminopyrimidine or STI571 or Imatinib mesylate (Buchdunger et al., 1996; Druker et al., 1996). Further studies demonstrated that Imatinib, beside BCR-ABL, is also able to inhibit platelet-derived growth factor receptor (PDGFR) and mast/stem cell growth factor receptor (SCFR), also known as proto-oncogene c-Kit, frequently mutated in gastrointestinal stromal tumor (GIST) (Buchdunger et al., 2000; Tuveson et al., 2001).

Several clinical trials have demonstrated the efficacy and safety of Imatinib in patients affected by CML, ALL, and GIST. In particular, Imatinib is able to induce the complete remission in CML patients refractory to other treatments. The phase III IRIS clinical trial confirmed the efficacy of Imatinib in patients with CML in chronic phase (Hahn et al., 2003). Other clinical trials were performed to assess the efficacy of Imatinib against c-KIT and PDGF receptor in patients affected by advanced and/or metastatic GIST tumor showing a good response rate and consequently an increased overall survival (OS) (Dagher et al., 2002; Blanke et al., 2008). After Imatinib discovery, some studies showed that a percentage of patients were resistant *ab initio* to treatment with Glivec due to the presence of specific mutated variants of BRC-ABL, while some patients developed resistances during treatment (Milojkovic and Apperley, 2009). To cope with the poor efficacy of Imatinib treatment in this category of patients, new second and third generation BCR-ABL inhibitors were developed, including Dasatinib (Sprycel®), Nilotinib (Tasigna®), Bosutinib (Bosulif®) and Ponatinib (Iclusig®) (Rossari et al., 2018).

The second class of small molecules directed to tyrosine kinase proteins was represented by Gefitinib (Iressa®) and Erlotinib (Tarceva®) both directed to the EGFR ATP-binding site and able to inhibit the abnormal activation of MAPK and PI3K/AKT pathways overexpressed in cancer cells (Nicholson et al., 2001; Yarden, 2001).

The first anti-EGFR agent approved in 2001 for the treatment of NSCLC was Gefitinib, a potent and selective inhibitor of both EGFR and HER-2 kinases (Barker et al., 2001). In preclinical studies, Gefitinib demonstrated antitumor activity in several human cancer cell lines over-expressing EGFR, including lung, ovarian, breast, and colon cancer cell lines (Ciardiello et al., 2000). Currently, Gefitinib is used for the treatment of NSCLC. The first clinical trials recorded a partial remission in 10–15% of patients with NSCLC (Kris et al., 2003) although it showed reduced efficacy when administered in combination with other chemotherapeutic agents. The second EGFR selective inhibitor was Erlotinib, with a mechanism of action similar to Gefitinib. Erlotinib is approved for the treatment of NSCLC and for advanced and/or metastatic pancreatic carcinoma, in association with gemcitabine. Both Gefitinib and Erlotinib do not induce complete remission but do increase OS rate and limit tumor growth (Steins et al., 2018).

Another specific inhibitor of HER1 and HER2, approved in 2007, is Lapatinib (Tiverb®), developed against HER2 receptors. Lapatinib is able to bind the ATP-binding site of the HER2 receptor intracellular domain resulting in the inhibition of tumor cell growth. Through the introduction of targeted therapy, with both Trastuzumab and Lapatinib, the poor prognosis of HER2-positive cancer patients has been significantly ameliorated (Slamon et al., 2001). Currently, based on numerous clinical trials, Lapatinib is used in association with several chemotherapeutic agents, such as capecitabine (Geyer et al., 2006) or trastuzumab (Blackwell et al., 2012) in patients with advanced HER2-positive breast cancer. Despite the important clinical results obtained with Lapatinib, today it is used as a third or fourth line treatment, after more effective treatments, such as Trastuzumab-emtansine, or Neratinib, another irreversible pan-tyrosine kinase inhibitor (Voigtlaender et al., 2018).

Another class of tyrosine kinase inhibitors is represented by VEGF inhibitors, able to inhibit also other receptors, such as PDGFR, KIT and FLT3. The first two chemical small molecules synthesized and directed to the ATP binding pocket of VEGF receptor were Sunitinib (Sutent®) and Sorafenib (Nexavar®) (Ivy et al., 2009).

As anti-angiogenic drugs, both Sunitinib and Sorafenib are widely used in tumors for which few effective treatments are available and in advanced diseases after the failure of standard chemotherapy (Herrmann et al., 2008; Ivy et al., 2009). In particular, Sunitinib has been approved for the treatment of Imatinib-resistant GIST, renal carcinoma and neuroendocrine pancreatic tumors, while Sorafenib is indicated for the treatment of hepatocellular carcinoma, renal cell carcinoma and thyroid carcinoma (Imbulgoda et al., 2014; Hasskarl, 2018).

At the beginning of 2006, both FDA (January 2006) and EMA (July 2006) approved Sunitinib malate for the treatment of Imatinib-resistant GIST and advanced renal cell carcinoma. As mentioned, Sunitinib appears to inhibit multiple receptor tyrosine kinases by interfering with the ATP binding site. In particular, Sunitinib inhibits the activity of VEGF receptors 1 and 2, Kit, PDGFR-α, and –β, Fms-like TK-3 (FLT3); colony-stimulating factor receptor type 1 and neurotrophic factor receptor 7. By this way, Sunitinib is able to modulate tumor growth directly, by inhibiting the activation of signal transduction, and indirectly, by preventing tumor neo-vascularization (Adams and Leggas, 2007). Several *in vitro* studies showed the efficacy of Sunitinib in different tumor types, including colon, NSCLC, glioma, melanoma, etc. (Mendel et al., 2003). However, clinical trials showed Sunitinib more effective in patients with renal cell carcinoma and in those with acute myelogenous leukemia with FLT3 mutations (Fiedler et al., 2005; Motzer et al., 2006).

A few years before the approval of Sunitinib, Sorafenib, a small chemical molecule able to inhibit VEGF and PDGF receptors, was approved by the regulatory agencies. This drug was initially developed as a direct inhibitor of RAF-1 and BRAF intracytoplasmic serine/threonine kinases. Sorafenib was initially developed in 2001 (approved in 2004) by Bayer Pharmaceuticals as a selective inhibitor of both mutated and wild-type RAF, and *in vitro* showed a strong inhibitory power toward MAPKs pathway regulated by RAF. Subsequently, an inhibitory activity was also demonstrated for VEGFR1/2 and PDFGR in several *in vitro* models (Wilhelm et al., 2004). Further phase I, II, and III clinical trials showed the efficacy of Sorafenib in renal cell carcinoma patients, hepatocellular carcinoma patients, and thyroid cancer patients thanks to the multiple inhibition of VEGFR, PDGFR and BRAF (Strumberg et al., 2005; Gupta-Abramson et al., 2008; Llovet et al., 2008).

Other anti-angiogenic drugs are Aflibercept and Pegaptanib sodium, respectively a chimeric protein and an aptamer both directed against VEGF (Lytvynchuk et al., 2015).

A class of selective small molecules completely different from those discussed so far is that of mTOR inhibitors. mTOR is an intracellular serine/threonine kinase that plays a fundamental role in the regulation of gene expression and in the progression of the cell cycle from G1 to S phase. The first mTOR inhibitor was rapamycin derived from *Streptomyces hygroscopicus*. The two drugs currently used in the field of oncology, Temsirolimus (Torisel®) and Everolimus (Afinitor®) were derived from rapamycin (Sirolimus) and are still used in different tumor forms, such as renal cell carcinoma, mantle cell lymphoma, breast cancer and neuroendocrine pancreatic carcinoma (Fasolo and Sessa, 2012). mTOR was discovered by studying the mechanism of action of rapamycin, a macrolide antibiotic discovered in 1975 (Vézina et al., 1975). Rapamycin anticancer activities were defined in the '90s when it was found that it inhibited cellular proliferation and cell cycle progression by blocking mTOR/mTORC1 complex (Jayaraman and Marks, 1993; Carew et al., 2011).

Temsirolimus, previously named cell cycle inhibitor-779, is a soluble ester of rapamycin, identified in the ‘90s and subsequently used as an anticancer agent (Peralba et al., 2003). Everolimus, previously named RAD001, is a derivative of rapamycin able to bind FKBP12 and inhibit the mTORC1 complex, resulting in the down-regulation of the PI3K signal transduction pathway, which is frequently activated in human malignancies (Hasskarl, 2018). Both drugs showed great efficacy in the clinical trials. Temsirolimus showed anti-tumor activities toward several preclinical tumor models and in phase I–III trials for advanced renal cell carcinoma and mantle cell lymphoma (Neshat et al., 2001; Yu et al., 2001; Hudes et al., 2007; Ansell et al., 2008). More recently, Everolimus also showed a great anti-tumor activity both *in vitro* and in preclinical tumor models (O'Reilly et al., 2010). Several clinical trials have tried to assess the efficacy of Everolimus in hematological and solid tumors and some of them have provided encouraging results for its clinical use (Hasskarl, 2018).

Among the selective serine/threonine kinase inhibitors, BRAF inhibitors (Vemurafenib and Dabrafenib) and MEK inhibitors (Trametinib and Cobimetinib) are widely used in clinical practice for the treatment of mutated *BRAF*^*V*600*E*^ melanomas, providing significant improvement in survival rates (Robert et al., 2015). Both Vemurafenib (Zelboraf® or PLX4032) and Dabrafenib (Tafinlar®) were approved after 2010 and are directed toward RAF protein, belonging to the RAS signal transduction pathway. The 15% of melanoma patients harbor RAS mutations and another 40–60% of patients are positive to the *BRAF*^*V*600*E*^ mutations. Therefore, the discovery of selective BRAF inhibitors represented a turning point in the management of this aggressive form of tumor. Both Vemurafenib and Dabrafenib induce melanoma cell apoptosis by interfering with the B-Raf/MEK/ERK pathway. Despite several clinical trials demonstrated the efficacy of these treatments in patients positive for *BRAF*^*V*600*E*^ mutation, resistance mechanisms limit the efficacy of the therapy in a high percentage of patients (Leonardi et al., 2018; Salemi et al., 2018).

To overcome these resistance mechanisms, BRAF inhibitors are generally combined with MEK inhibitors, such as Trametinib (Mekinist®) and/or Cobimetinib (Cotellic®) (Robert et al., 2015; Ascierto et al., 2016). Both Trametinib and Cobimetinib were approved in the last 5 years and are indicated for the treatment of BRAF mutant metastatic melanoma in order to avoid tumor relapse after surgical excision (Long et al., 2017).

Other selective inhibitors were developed to inhibit the proteasome machinery for the treatment of hematological malignancies, especially for multiple myeloma and mantle cell lymphoma. Among these inhibitors, Bortezomib (Velcade®) and Carfilzomib (Kyprolis®) are used in clinical practice; thanks to the inhibition of proteasome they prevent the degradation of pro-apoptotic factors, thus favoring the apoptotic death of cancer cells (Manasanch and Orlowski, 2017; Goldschmidt et al., 2018).

All these targeted therapeutic agents are currently used for the treatment of tumors, often in combination with other standard chemotherapeutic agents or in combination with monoclonal antibodies and/or other selective inhibitors (Vanneman and Dranoff, 2012). The availability of more drugs directed to different molecular targets has stimulated the development of different therapeutic strategies to make treatments more effective and to overcome possible innate or acquired pharmacological resistance (Mokhtari et al., 2017).

## Immune checkpoint inhibitors as a new strategy for cancer treatment

Cancer immunotherapy has experienced remarkable advances in recent years. After 2010, new monoclonal antibodies directed toward tumor antigens or T-cell protein receptors that downregulate the immune response have been developed (Haanen and Robert, 2015). These new drugs are defined immune checkpoint inhibitors and are monoclonal antibodies anti-cytotoxic T-lymphocyte-associated antigen 4 (anti-CTLA4) and anti-programmed cell death protein 1 antibody (anti-PD1), located in the membrane surface of T-cell and cancer cells, respectively (Seidel et al., 2018).

The first approved immune checkpoint inhibitor was Ipilimumab (Yervoy®), in 2011. This is a human IgG1 antibody that binds the membrane protein CTLA-4 expressed in regulatory T cells. The tumor microenvironment is able to induce the overexpression of CTLA-4 that binds the stimulating protein CD80 and CD86 present in the antigen presenting cells preventing their interaction with the T-cell surface receptor, responsible for the activation of immune system against cancer cells (Zitvogel et al., 2013).

Currently, Ipilimumab is used alone or in combination with Nivolumab for the treatment of unresectable or metastatic melanoma. The first clinical trials reported the improvement of long term-survival in melanoma patients with prolonged PFS and OS (Amdahl et al., 2016). Furthermore, several clinical trials are currently underway to establish the therapeutic efficacy of Ipilimumab, alone or in combination with Nivolumab, in other tumors as well as NSCLC, prostate cancer, renal cell carcinoma, etc. (Sakamuri et al., 2018).

More recently, two immune checkpoint inhibitor monoclonal antibodies were approved for the treatment of NSCLC, metastatic melanoma, NHL, and urothelial carcinoma as long as these tumors are positive to the presence of PD-L1. These two inhibitors are Nivolumab (Opdivo®) and Pembrolizumab (Keytruda®). Both drugs are human IgG4 anti-PD-1 antibodies directed toward the programmed cell death 1(PD-1) receptor of lymphocytes. This receptor, when linked to the PD-L1 antigens expressed from some tumors, is a down-regulator of T-cells, which become unable to recognize and kill cancer cells.

NSCLC patients treated with Nivolumab showed a lower risk of death and higher median PFS and OS compared to NSCLC patients treated with docetaxel (3-years PFS rate of 10% compared to <1%; 3-years OS rate 17 vs. 8% in patients treated with docetaxel; Vokes et al., 2018). These encouraging results are also confirmed by other clinical trials (Ramos-Esquivel et al., 2017).

Pembrolizumab has also shown therapeutic effects in patients with metastatic tumors, with limited side effects. Pembrolizumab is comparable to Nivolumab, suggesting a possible use of these two drugs as a first-line treatment for advanced or metastatic tumors (Brahmer et al., 2017; Fessas et al., 2017; Frenel et al., 2017).

Durvalumab (Imfinzi®), a human IgG1κ monoclonal antibody, blocks the interaction of programmed cell death ligand 1 (PD-L1) with the PD-1 and CD80 receptors. Durvalumab is approved for the treatment of patients with locally advanced or metastatic urothelial carcinoma (Faiena et al., 2018). Several studies are also evaluating its use for the treatment of patients with NSCLC (Antonia et al., 2017).

Finally, the immune checkpoint inhibitors are often administered in combination with each other or in combination with other chemotherapeutic agents, in order to make the treatment as effective as possible and to prolong the PFS and OS of the patients. The combination of anti-PD1 and anti-CTLA-4 inhibitors has shown a more durable response compared to monotherapy (Mahoney et al., 2015). The use of these drugs has revolutionized the treatment of incurable tumors, such as metastatic melanoma and NSCLC, increasing the life expectancy of patients and counteracting the onset of new metastases.

## The role of molecular radiotherapy in cancer

Molecular radiotherapy (MRT), called also unsealed source radiotherapy or unsealed source radionuclide therapy, is a well-known therapeutic approach used in clinical practice since many decades, based on the use of radioactive compounds (radiopharmaceuticals). Generally, radiopharmaceuticals are administered by ingestion or injection and their action is expressed toward the target cells recognized by specific carrier or depends on the radioisotope properties. The first report of the use of radiopharmaceuticals dates back to 1942 when Hertz used iodine-131 as a treatment for the autoimmune Basedow-Graves disease (Hertz et al., 1942). Nowadays, MRT is used for the treatment of both cancer and benign diseases by using simple radioactive compounds (e.g., sodium iodide) or recombinant antibodies labeled with radionuclides, specific for certain cells and tissues (Volkert and Hoffman, 1999; Buscombe and Navalkissoor, 2012).

MRT could also be considered a type of targeted therapy for the treatment of specific areas through the biological and radiopharmaceutical properties of the radiation treatment (Jadvar, 2017). In particular, the administration of 131I-Sodium Iodide for the treatment of thyroid cancers and 89Sr-Strontium chloride and 32P-Sodium phosphate for the treatment of bone metastasis are well-recognized treatments used since 1978 (Kutzner et al., 1978). In contrast to external beam radiotherapy, the use of systemic radiopharmaceuticals specifically localizes primitive and metastasized cancer cells, widely disseminated in the whole body, with minimal radiation-related damage to normal tissues (Choi, 2018). Since the 1980s, several radiopharmaceuticals were developed for treatment of cancers. These drugs were used alone or in combination with molecular carriers for enhancing their specificity toward cancer cells (Wilbur et al., 1996; Zhu et al., 1998). Thanks to the technological advances in the fields of molecular biology, genetic engineering and chemistry it was possible to realize several conjugated drugs widely used in clinical practice. Among these, Iodine-131, MIBG (131I-MIBG metaiodobenzylguanidine), Radium-223 chloride, Strontium-89 chloride, Samarium-153 EDTMP, Phosphorus-32, Yttrium-90, and Yttrium-90 spheres were the most used drugs for both therapeutic and palliative purposes (Guerra Liberal et al., 2014; Jadvar, 2017).

Iodine-131 represents the first and most common radiopharmaceutical agents used for the treatment of thyroid cancers (Chung et al., 2010). It is composed by sodium iodide with a radioactive isotope of iodine. Its mechanism of action is based on the great affinity and uptake of iodide ion for the thyroid gland. This treatment is not only used for thyroid cancer pathologies but also for benign disease where the radiation emitted by radioiodine can have a beneficial effect (Silberstein et al., 2012). The beta radiations produced by sodium iodide determine damages to both normal and cancer thyroid cells inducing the cell deaths and thus having a therapeutic effect (Spitzweg et al., 2001).

Other unsealed radioactive sources are used as palliative treatments for the management of bone metastasis. Among these radioactive sources, Radium-223 chloride, Strontium-89 chloride, and Samarium-153 EDTMP are used for secondary bone metastatic disseminations of different cancer histotypes (Janjan, 1997; Choi, 2018). In particular, Strontium and Radium radioisotopes are taken up by bone as they mimic calcium ion, while samarium thanks to its covalent bond to tetraphosphate EDTMP is actively absorbed by osteoblasts, involved in the bone repair near the bone metastasis lesions (Wissing et al., 2013; Anderson et al., 2014). In these ways, Radium-223 chloride, Strontium-89 chloride, and Samarium-153 EDTMP can effectively counteract the progression of bone metastases, reduce patient suffering and prolong life expectancy.

Finally, other radiopharmaceuticals are used for the treatment of cancer, including Phosphorus-32, Yttrium-90 spheres for the treatment of colorectal liver metastasis, 131I-MIBG meta-iodo-benzylguanidine for the treatment of phaeochromocytoma and neuroblastoma, and Yttrium-90 and Lutetium-177 for the treatment of neuroendocrine tumors (Forrer et al., 2005; Sudbrock et al., 2010; Hadaki et al., 2011; Cheng et al., 2015).

## New frontiers in the treatment of cancer

The drugs discovery in oncology is a constantly evolving field and every year several new approaches are proposed. As discussed above, after the Second World War there has been a rapid growth in the number of drugs available thanks to the important discoveries obtained in the biological, genetic and molecular fields. Parallel to the increase in the number of available drugs, there was also an increase in the effectiveness of the treatments, which consequently led to a significant improvement in the survival and quality of life of the patients.

Many clinical trials are currently underway to develop new drugs and therapeutic approaches for the treatment of hematological tumors and solid tumors. In particular, important results were obtained in the field of cell therapy, with the implementation of the so-called CAR-T cell therapy (Chimeric-Antigen Receptor) which led to the recent approval of two treatments, axicabtagene ciloleucel (Yescarta®) and tisagenlecleucel (Kymriah®), used respectively for the treatment of patients with relapsed/refractory diffuse large B-cell lymphoma (DLBCL) and patients with relapsed/refractory B-cell precursor acute lymphoblastic leukemia (ALL) (Grupp, 2018).

CAR-T cell therapy consists in the ligation of engineered receptors to immune T cell specific for antigens expressed by the cancer cells, therefore the resulting chimeric T cell harbor a kind of monoclonal antibody with high specificity only toward cancer cells. In particular, the chimeric receptor is added to the immune cells by inserting a genic construct into the T cell DNA. The adjective “chimeric” means that the artificial receptors are constituted with protein structure derived from different DNA organisms and sources (Xu et al., 2018).

The realization of CAR-T cell therapy involves the removal of T-cells from the patient and their *in vitro* genetic modification for the addition of the chimeric receptor; subsequently, the engineered T-cells are reinfused into the patient, where they selectively interact with cancer cells, inducing immune-mediated cell death without affecting normal cells (Srivastava and Riddell, 2015). Potentially, CAR-T therapy can be implemented for all tumor types, a fundamental step for this possibility is to recognize specific antigens expressed by different tumors.

The first successful CAR-T therapy was developed against malignant B-cell responsible for a plethora of hematological tumors including acute ALL, chronic lymphocytic leukemia (CLL), and many different forms of Hodgkin's lymphoma. The axicabtagene ciloleucel (Axi-cel) therapy consist of chimeric T cells receptors against CD19, a surface molecule expressed in B-cells after their differentiation. Therefore, Axi-cel is effective for both normal and malignant B-cells determining their cell death, however, the B-cell precursor does not present CD19 antigen and for this reason is not affected by the treatment allowing the reconstitution of normal B-cells after treatment (Lee et al., 2015).

In 2010, Kochenderfer treated the first patient with an anti-CD19 CAR T therapy and obtained a significant clinical response (Kochenderfer et al., 2010). Subsequently, several clinical trials were performed to assess the efficacy and safety of anti-CD19 CAR-T. The most important clinical trial is the ZUMA-1 conducted on 22 patients with aggressive B cell lymphomas that showed an overall response rate in 73% of patients and a complete response in 55% (Kochenderfer et al., 2017). These results were confirmed by the phase II ZUMA-1 trials that demonstrated an overall response rate and a complete response in 82 and 54% of 101 total patients, respectively. However, some of these patients experienced acute toxicities and some of them died during the treatment (Neelapu et al., 2017).

The other currently approved CAR-T therapy for the treatment of B-cell acute lymphoblastic leukemia is the one using tisagenlecleucel. Similar to Axi-cel therapy, tisagenlecleucel also relies on artificial T cells with a chimeric anti-CD19 antigen. This therapy was developed by Carl June at the University of Pennsylvania and is a personalized treatment for the patient that is obtained with a 22-days experimental procedure. Via viral vectors, the patient's T cells are modified by adding a chimeric gene coding for the specific CAR receptor for leukemic cells (Porter et al., 2011).

The clinical trials showed that CLL and ALL patients treated with Kymriah had a promising and durable antitumor efficacy with an 82% overall response rate and a complete response in 68% of patients (Mueller et al., 2017). Treatment with tisagenlecleucel is also associated with a series of adverse effects, among which the most important are the cytokine release syndrome and neurological events that require treatment in specialized centers (Badieyan and Hoseini, 2018). Many studies are trying to apply CAR-T therapy to solid tumors using modified heterologous cells obtained in cell factories.

Recently, many researchers are trying to develop new therapeutic approaches, based on genomic editing using CRISPR/Cas9 technology to correct genetic aberrations responsible for neoplastic transformation (Zhan et al., 2018).

Finally, in recent years, many research centers are developing therapeutic anticancer vaccines designed according to the individual characteristics of the tumor to make the immune system more active against the cancer cells and determine their death. However, the realization of these vaccines is complex due to the variability that characterizes each tumor. Already in 2008, the Oncophage vaccine was approved for the treatment of glioma, renal cancer, and metastatic melanoma; this vaccine consists of the heat shock protein 96 extracted directly from the tumor tissue and is supposed to stimulate the immune response against neoplastic cells of the same tumor (di Pietro et al., 2008). Subsequently, in 2010, another vaccine was approved, sipuleucel-T, for the treatment of metastatic, hormone-refractory, prostate cancer. This vaccine is again produced for each individual patient and consists of pulsed patient's dendritic cells with recombinant prostatic acid phosphatase expressed in the 95% of prostate cancer cells. In this way, the administration of the vaccine induces an increase in the immune response directed only to the tumor cells, determining their elimination (So-Rosillo and Small, 2006). Many other anticancer therapeutic vaccines are under study, but production difficulties make this approach particularly expensive and not suitable for all patients.

In conclusion, it is clear that cancer drug treatments are constantly evolving. From the Second Post-War to the advent of the new millennium, there has been an increase in the number of drugs and therapies available for the treatment of all hematological and solid tumors that have contributed to the significant reduction in cancer mortality rates. Furthermore, thanks to the primary and secondary prevention campaigns the reduction of incidence rates was recorded for many tumors, particularly for those of predominantly environmental etiology (Figure 1).

In the next few years, the development and approval of new highly innovative chemical, biological and biotechnological drugs are expected. These new treatments will start a new revolution in the field of clinical oncology, mainly based on a specific individual approach for each patient, a new personalized and more effective medicine.

## Author contributions

LF and SS wrote the manuscript and were involved in data collection. LF has made the figures and tables. SS and ML conceived and reviewed the final version of the manuscript. All authors read and approved the final version of the manuscript.

### Conflict of interest statement

The authors declare that the research was conducted in the absence of any commercial or financial relationships that could be construed as a potential conflict of interest.
